# Stent-retriever assisted vacuum-locked extraction (SAVE) versus a direct aspiration first pass technique (ADAPT) for acute stroke: data from the real-world

**DOI:** 10.1186/s12883-019-1291-9

**Published:** 2019-04-15

**Authors:** Alex Brehm, Volker Maus, Ioannis Tsogkas, Ruben Colla, Amélie Carolina Hesse, Roland Gerard Gera, Marios-Nikos Psychogios

**Affiliations:** 1 Department of Neuroradiology, University Medical Center Goettingen, Robert-Koch-Str. 40, 37075 Göttingen, Germany; 2 Department of Medical Statistics, University Medical Center Goettingen, Humboldtallee 32, 37073 Göttingen, Germany; 3 Department of Neuroradiology, Clinic of Radiology & Nuclear Medicine, University Hospital Basel, Basel, Switzerland

**Keywords:** Acute ischemic stroke, Thrombectomy, Large vessel occlusion, Technique

## Abstract

**Background:**

Embolectomy is the standard of care in acute ischemic stroke (AIS) caused by large vessel occlusion (LVO). Aim of this study was to compare two techniques: A Direct Aspiration First Pass Technique (ADAPT) and Stent-retriever Assisted Vacuum-locked Extraction (SAVE) stratified by the occluded vessel.

**Methods:**

One hundred seventy-one patients (71 male) treated between January 2014 and September 2017 with AIS due to LVO of the anterior circulation (55 carotid T, 94 M1, 22 M2) were included. Treatment techniques were divided into two categories: ADAPT and SAVE. Primary endpoints were successful reperfusion (mTICI ≥2b), near-perfect reperfusion (mTICI ≥2c) and groin puncture to reperfusion time. Secondary endpoints were the number of device-passes, first-pass reperfusion, the frequency of emboli to new territory (ENT), clinical outcome at 90 days, and the frequency of symptomatic intracranial hemorrhage (sICH). Analysis was performed on an intention to treat basis.

**Results:**

Overall, SAVE resulted in significant higher rates of successful reperfusion (mTICI≥2b) compared to ADAPT (93.5% vs 75.0%; *p* = 0.006). After stratification for the occluded vessel only the carotid T remained significant with higher rates of near-perfect reperfusion (mTICI≥2c) (55.2% vs 15.4%; *p* = 0.025), while for successful reperfusion a trend remained (93.1% vs 65.4%; *p* = 0.10). Groin to reperfusion times were not significantly different. Secondary analysis revealed higher rates of first-pass successful reperfusion (59.6% vs 33.3%; *p* = 0.019), higher rates of first-pass near-perfect reperfusion in the carotid T (35.4% vs 16.7%; *p* = 0.038) and a lower number of device-passes overall (median 1 IQR 1–2 vs 2 IQR 2–3; *p* <  0.001) and in the carotid T (median 2 IQR 1.3 vs 3 IQR 2–5; p <  0.001) for SAVE. Clinical outcome and safety parameters were comparable between groups.

**Conclusions:**

Embolectomy using SAVE appears superior to ADAPT, especially for carotid T occlusions with regard to reperfusion success.

## Background

Since multiple large randomized clinical trials demonstrated the superiority of endovascular treatment (EVT) over best medical treatment in patients with acute ischemic stroke (AIS) caused by large vessel occlusion (LVO) of the anterior circulation [1–5], research priorities shifted among other things to improving angiographic outcomes. However several technical approaches were published in the recent years; they can generally be divided into three major categories: a direct aspiration first-pass technique (ADAPT), the primary use of a stent-retriever with Balloon-Guiding Catheter (BGC) and the primary combined approach with simultaneous intracranial use of an aspiration-catheter and a stent-retriever [6] with or without additional use of a BGC.

While a recent randomized controlled study comparing the first two techniques showed no superiority of either one in terms of reperfusion scores (mTICI) and clinical outcome (90 days mRS) [7], smaller observational studies showed very high reperfusion rates and excellent functional outcome for combined approaches such as “Continuous aspiration prior to intracranial vascular embolectomy” (CAPTIVE), “Aspiration-Retriever Technique for Stroke” (ARTS) and “Stent-retriever Assisted Vacuum-locked Extraction” (SAVE) [8–10]. This notion is supported by an in-vitro comparison of stent-retriever plus aspiration and stent-retriever alone, in which the combined approach showed significantly higher rates of successful revascularization [11]. Regarding the additional use of a BGC, the “PRoximal balloon Occlusion TogEther with direCt Thrombus aspiration during stent retriever thrombectomy” (PROTECT) and PROTECT plus study showed very promising results [12, 13]. This is in line with in vitro work performed by Chueh et al., showing a reduction in distal emboli due to proximal flow control [14].

To determine, if a combined approach using SAVE is more efficient than ADAPT, we compared the angiographic results from our center on an intention to treat basis.

## Methods

### Study population

Our prospectively acquired stroke database was screened for all patients who underwent EVT either with the ADAPT or the SAVE technique for LVO in the anterior circulation (carotid T, middle cerebral artery in the M1 and M2 segment) between January 2014 (after the introduction of large bore aspiration-catheters) and September 2017. In total, 171 patients were included; of those, 50 patients were treated with ADAPT, 22 with ADAPT plus stent- retriever rescue, 97 with SAVE and 2 patients with SAVE plus aspiration rescue. Patients with tandem occlusions (*n* = 34) were, in contrast to the initial SAVE paper and the ASTER trial, not excluded from our study. 118 (69.0%) patients received intravenous tissue plasminogen activator (ivTPA). There was no difference in the administration of ivTPA between both groups (ADAPT 69.4%, SAVE 68.7%; *p* = 0.916) Consent for treatment from the patients was obtained according to international guidelines. The study was approved by the local institutional review board. The need for an additional formal application or separate consent regarding the inclusion in our retrospective study was waived. The study was conducted in accordance with the Declaration of Helsinki.

### Image analysis

The angiograms were evaluated blinded to all clinical data by two neuroradiologists with different experience (I.-T. > 5 years and R. C. > 3 years). The percentage of reperfusion was based on PA and lateral view images on the final angiograms. The images were then rated according to the definition of the revised mTICI score [15] including mTCI 2c (> 90% reperfusion) [16]. Cases, in which the terminal segment of the internal carotid artery (ICA) was occluded plus/minus an occlusion of the A1 and/or M1 segment, were classified as carotid T occlusions. An M1 occlusion was defined as an isolated occlusion of the proximal and/or distal A. cerebri media. The M2 segment was defined as the segment after the horizontal segment of the M1. In case of an M1 or M2 occlusion, every other occlusion after the intervention, which was not in the affected media territory, was rated as an emboli to new territory (ENT). In patients with a carotid T occlusion, every occlusion, which was not in the affected media or anterior territory, was rated as an ENT. If different ratings occurred the angiograms were reevaluated in a joint session until consensus was reached. These ratings were used for further analysis. The ASPECT and Collateral scores were rated independent from the intervention by an intern and an attending (A.C. H. > 2 years and I.-T. > 5 years). CT datasets for rating ASPECT and Collateral scores were available for all patients. For rating collaterals the single phase Menon score was used. This score evaluates the anterior-media and media-posterior collaterals separately on a 5 point scale. Therefor the maximum value is 2 × 5 = 10 (increased or normal collaterals compared to the asymptomatic hemisphere) and the minimum value is 0 (absent collaterals) [17]. Intracranial hemorrhages were graded after the ECASS criteria.

### Thrombectomy technique

The ADAPT technique and the SAVE technique have been described previously [10, 18]. In case of ADAPT an 8F Mach1 (Boston Scientific, Marlborough, MA, USA), an 8F Vista Brite Tip (Codman, Raynham, MA, USA) or a NeuronMAX 088 (Penumbra, Alameda, CA, USA) were used in more than 97% of the cases as guide-catheters, while as aspiration catheter the SOFIA 6F (Microvention, Tustin, CA; USA) was used in 27,8%, the SOFIA plus (Microvention) in 15.4%, the SOFIA 5F (Microvention) in 14.3%, the CATALYST 6 (Stryker, Neurovascular, Mountain View, CA, USA) in 10.6%, the 5MAX ACE (Penumbra) in 10.6%, the ACE 68 (Penumbra) in 7.1%, the 5MAX (Penumbra) in 5.9%, the 4 MAX (Penumbra) in 5.9%, the CATALYST 5 (Stryker) in 1,2% and the ACE 64 (Penumbra) in 1.2% of the cases. In case of incomplete reperfusion it was at the physicians discretion to stick with the ADAPT plan or switch to a rescue maneuver, which incorporates a stent-retriever. All patients treated with ADAPT as a first-pass technique were assigned to group 1 regardless of the need of a rescue maneuver. In case of SAVE, an 8F Mach1 (Boston Scientific), an 8F Vista Brite Tip (Codman) or a NeuronMAX 088 (Penumbra) were used in more than 97% of the cases as guide-catheters, while the Trevo XP ProVue Retriever (Stryker) was used in 89.8%, the pRESET (Phenox, Bochum; NRW; Germany) in 5.6%, the APERIO Thrombectomy device (ACANDIS, Pforzheim, B-W; Germany) in 2.8% and the 3D Seperator (Penumbra) in 1.8% of the cases as a stent-retriever. As aspiration-catheters the CATALYST 6 (Penumbra) was used in 35.8%, the SOFIA 6F (Microvention) in 17.0%, the SOFIA 5F (Microvention) in 15.1%, the 5MAX ACE (Penumbra) in 10.4%, the ACE 68 (Penumbra) in 9.4%, the 5MAX (Penumbra) in 5.7% the CATALYST 5 in 3.8% and the 4 MAX in 2.8% of the cases. In case of incomplete perfusion physicians were allowed to switch to a rescue maneuver. All cases treated with SAVE as a first-pass technique were assigned to group 2. In case of a tandem occlusion the proximal ICA occlusion was passed with the microcatheter and over the microcatheter the stent-retriever was advanced to the distal occlusion. After deploying the stent-retriever, stenting and/or balloon angioplasty of the proximal occlusion was performed. In the next step the SAVE maneuver was performed at the distal occlusion. 72% of the tandem occlusions were stented primarily, while 12% of the tandem occlusions received balloon angioplasty only, the remaining 16% were not treated primarily.

### Statistical analysis

The aim of this observational, one-center study is the comparison of the ADAPT with the SAVE technique. The assignment to treatment was not done prospectively. It was at the discretion of the treating physician to decide which technique to use. However, most ADAPT patients were recruited between 2014 and the end of 2016, while most recent cases were treated with the SAVE technique. The analysis is based on the ITT population. Therefore, we have two groups: first-pass ADAPT (group 1) and first-pass SAVE (group 2). The primary hypothesis is that the SAVE technique is superior to the ADAPT technique. Two co-primary end-points where assessed to test this hypothesis: higher frequency of successful/near-perfect reperfusion (defined as mTICI ≥2b and ≥ 2c); and non-inferiority of the time from groin puncture to successful reperfusion, with a non-inferiority threshold of 1200 s (20 min). Decision on the non-inferiority threshold was made prior to the analysis of the data.

Secondary endpoints were first-pass successful/near-perfect reperfusion (defined as mTICI ≥2b or ≥ 2c), clinical outcome measured by 90 days modified Rankin Scale (mRS), the number of device-passes, the frequency of ENT and symptomatic intracranial hemorrhages (sICH). The analysis was performed for the anterior circulation and stratified by the occluded vessel (i.e. carotid T, M1 segment, M2 segment).

Continuous study parameters were visualized using grouped boxplots and Q-Q plots. To examine differences of the study parameters between treatment groups, t-tests were conducted on each parameter if normality could be assumed, otherwise Mann-Whitney-Tests were performed. Categorical parameters were compared using the fisher’s exact test.

A multivariate logistic regression using covariates on the co-primary endpoint “successful/near-perfect reperfusion” was performed. The two thrombectomy techniques were included as essential variables as well as treatment location with carotid T, M1 and M2, while baseline variables which tend to differ between groups (defined as *p* <  0.2) as well as treatment location interactions were used as additional covariates. To prove non-inferiority for the endpoint “groin to reperfusion time”, pairwise location shifts with corresponding confidence intervals (CI) are calculated utilizing Hodges-Lehmann estimators.

## Results

One hundred and seventy-one patients (71 male, 41.5%) were included in our study. Baseline characteristics are depicted in Table 1. Of the 171 included patients, 72 (42.1%) were assigned to the ADAPT group and 99 (57.9%) to the SAVE group. In our sample only dyslipidemia (*p* = 0.146) and time from admission to reperfusion (*p* = 0.045) were sufficiently different between both groups (see Table 1). Shorter admission to reperfusion times in the SAVE group are due to more cases triaged with a new one-stop management paradigm [19]. Regarding further analyses it is noteworthy that 30.6% (22/72) of the ADAPT cases needed an additional stent-retriever rescue (Table 1), while only 2% (2/99) of the SAVE cases required an additional rescue maneuver. Vessel occlusion location was balanced with carotid T occlusion, M1 and M2 occlusion in 36.1, 50 and 13.9% of the ADAPT cases and 29.3, 58.6 and 12.1% of the SAVE cases (*p* = 0.530). The results of the multivariate logistic regression are visualized in Table 2. The *p*-values were adjusted using the Bonferroni method. Using the joint test the impact of the applied technique on successful reperfusion (mTICI≥2b) was highly significant with a p-value of 0.006 and an Odds ratio (OR) of 7.231. Regarding near-perfect reperfusion (mTICI ≥2c) the joint test approached significance with a p-value of 0.052. However the OR of 2.738 indicates a relevant difference and that the failure to show significance is only due to the small sample size. The co-variate “time from admission to reperfusion” had a significant effect on recanalization with an OR of 0.988 (*p* = 0.033) for mTICI ≥2b and 0.986 (*p* = 0.003) for mTICI ≥2c as well. Although the OR for the covariate “time from admission to reperfusion” is very low, it has to be noted that it is an incremental per minute change [20].

**Table 1:** Baseline Features of the study population

Variable*	Overall (*n* = 171)	ADAPT (*n* = 72)	SAVE (*n* = 99)	*p* Value
Site of occlusion				0.530
Carotid T	55 (32.2)	26 (36.1)	29 (29.3)	
M1 segment	94 (55.0)	36 (50.0)	58 (58.6)	
M2 segment	22 (12.8)	10 (13.9)	12 (12.1)	
Age (mean, SD)	73.7 ± 12.6	72.6 ± 14.1	74.5 ± 11.45	0.314
Male sex	71 (41.5)	30 (41.7)	41 (41.4)	1
Arterial Hypertension	137 (81.1)	60 (84.5)	77 (78.6)	0.608
Dyslipidemia	66 (40.0)	33 (46.5)	33 (35.1)	**0.146**
Diabetes mellitus	49 (29.7)	18 (25.7)	31 (32.6)	0.571
Atrial fibrillation	78 (47.3)	35 (49.3)	43 (45.7)	1
Peripheral artery occlusive disease	14 (8.4)	6 (8.6)	8 (8.3)	0.608
NIHSS on admission (Median, IQR)	16, 10–20	16, 9–20	16, 12–20	0.336
CCT-ASPECTS (Median, IQR)	8, 7–9	8, 7–9	8 7–9	0.632
Tandem occlusion	34 (19.9%)	14 (19.4%)	20 (20.2%)	1
Rescue maneuver	24 (14.0)	22 (30.6)	2 (0.02)	
Collaterals (Median, IQR)	6, 4–8	6, 5–8	6, 5–7	0.343
Mothership patients	124 (63.9)	61 (70.1)	26 (29.9)	
Symptom to admission (Mean, SD)	135.7 ± 102.7	124.2 ± 83.0	145.0 ± 115.9	0.233
Admission to reperfusion (Mean, SD)	105.9 ± 43.3	110.8 ± 39.33	96.68 ± 41.31	**0.045**
Symptom to reperfusion (Mean, SD)	237.9 ± 103.4	232.95 ± 81.7	241.7 ± 117.8	0.624
ivTPA	118 (69.0)	50 (69.4)	68 (68.7)	0.916
Interventionalist 1	5 (2.9)	2 (2.8)	3 (3.0)	
Interventionalist 2	67 (39.2)	17 (23.6)	50 (50.5)	
Interventionalist 3	29 (17.0)	23 (31.9)	6 (6.1)	
Interventionalist 4	22 (12.9)	20 (27.8)	2 (2.0)	
Interventionalist 5	5 (2.9)	4 (5.6)	1 (1.0)	
Interventionalist 6	21 (12.3)	3 (4.2)	18 (18.2)	
Interventionalist 7	19 (11.1)	3 (4.2)	16 (16.2)	
Interventionalist 8	3 (1.8)	0 (0.0)	3 (3.0)	

*Data are presented as numbers (%) unless otherwise specified; *ASPECTS* Alberta stroke programme early CT score, *ICA* Internal Carotid Artery, *IQR* interquartile range, *NIHSS* National Institutes of Health Stroke Scale, *SD* standard deviation, *CCT* cranial computer tomography; Variables which tend to be different (*p* < 0.2) are highlighted, as they are used as Covariantes in the primary multivariante logistic regression

**Table 2:** Results from the primary multivariate logistic regression

	Goal	Comparison	Vessel	OR	*p* Value
Technique	mTICI ≥2b	SAVE vs ADAPT	Joint Test	7.231	**0.006**
			Carotid T	11.109	0.100
			M1	8.325	0.187
			M2	4.089	1
	mTICI ≥2c	SAVE vs ADAPT	Joint Test	2.738	0.052
			Carotid T	6.247	**0.025**
			M1	1.326	1
			M2	1.655	1
Vessel	mTICI ≥2b		Joint Test		0.547
	mTICI ≥2c		Joint Test		0.101
Vessel * Technique	mTICI ≥2b		Joint Test		0.864
	mTICI ≥2c		Joint Test		0.171
Admission to reperfusion	mTICI ≥2b	Per minute	Global	0.988	**0.033**
	mTICI ≥2c	Per minute	Global	0.986	**0.003**
Dyslipidemia	mTICI ≥2b	Yes vs no	Global	0.452	0.164
	mTICI ≥2c	Yes vs no	Global	0.809	0.555

*mTICI* modified thrombolysis in cerebral infarction score

After stratification for the occluded vessel this effect remained significant for the carotid T (*n* = 55). The OR (SAVE vs ADAPT) for achieving near-perfect reperfusion defined as mTICI≥2c was 6.247 (*p* = 0.025) and 11.109 (*p* = 0.10) for mTICI≥2b respectively. The large OR in case of mTICI≥2b indicates that significance was not achieved due to a low sample size. For the M1 segment no significant effect was observed, however the OR for archiving mTICI≥2b was 8.325 (*p* = 0.187). For the M2 segment (*n* = 22) no difference in reperfusion success is observable in this sample. All reperfusion rates and OR are shown in Tables 2 and 3. A mean groin to reperfusion time of 55.6 min (± 31.3 min) was measured in the ADAPT group while 52.1 min (± 25.3 min) were documented in the SAVE group. The mean groin to reperfusion time for ADAPT is prolonged to 72.63 min (± 26.45 min), if a rescue maneuver was necessary compared to 50.07 min (± 35.89 min; *p* = 0.009) if not. The calculated CIs for the location shift were all between − 20 and + 20 min. Therefor non-inferiority can be assumed. For the carotid T the mean groin to reperfusion times were 61.1 min (±25.9 min) and 73.7 min (± 32.9 min; *p* = 0.129), for the M1 segment 47.7 min (± 23.2 min) and 44.6 min (±25.8 min; *p* = 0.559) and for the M2 segment 51.8 min (±29.6 min) and 49.4 min (±26.2 min; *p* = 0.856) for SAVE and ADAPT respectively. For all vessels non-inferiority can be assumed.

**Table 3:** Reperfusion results and time-metrics

Endpoint	Overall	ADAPT	SAVE	*p* Value
mTICI≥2b	86.0%	75.0%	93.5%	**0.006**
Carotid T	80.0%	65.4%	93.1%	0.100
M1 segment	90.4%	83.3%	94.8%	0.187
M2 segment	81,8%	70.0%	91.7%	1
mTICI≥2c	45.6%	33.3%	54.5%	0.052
Carotid T	36.4%	15.4%	55.2%	**0.025**
M1 segment	55.3%	50%	58.6%	1
M2 segment	27.3%	20.0%	33.3%	1
mTICI3	25.7%	22.2%	28.3%	1
Carotid T	16.4%	11.5%	20.7%	1
M1 segment	33.0%	32.8%	33.3%	1
M2 segment	18.2%	10%	25%	1
Groin to reperfusion (Mean, SD)	55.2 ± 29.2	55.6 ± 31.3	52.1 ± 25.3	0.432
Carotid T	66.9 ± 29.7	73.7 ± 32.9	61.1 ± 25.9	0.129
M1 segment	46.5 ± 24.1	44.6 ± 25.8	47.7 ± 23.2	0.559
M2 segment	50.8 ± 27.6	49.4 ± 26.2	51.8 ± 29.6	0.856

*mTICI* modified thrombolysis in cerebral infarction score, *SD* standard deviation

The secondary endpoints are documented in Table 4. The *p*-values were adjusted using the Bonferroni method. For the anterior circulation SAVE resulted in a significant higher rate of first-pass mTICI ≥2b (59.6% vs 33.3%; *p* = 0.019) and a non-significant higher rate of first-pass mTICI ≥2c (35.4% vs 16.7%; *p* = 0.171) reperfusion compared to ADAPT. In the carotid T SAVE resulted in a significant higher rate of first-pass mTICI ≥2c (31.0% vs 0%; *p* = 0.038). In the M1 and M2 segment this effect was not observed. No effect on 90 days mRS was observed in our sample (median 3, Interquartile Range (IQR) 2–5 vs median 3, IQR 1–6; *p* = 1) over all vessels. The techniques showed no effect on the frequency of sICH (4.2% vs 5.2%; p = 1) or postinterventional subarachnoid hemorrhages (20.8% vs 12.1%, p = 1). Significantly less device-passes overall (median 1, IQR 1–2 vs median 2, IQR 2–3; *p* <  0.001) were documented for the SAVE technique. The number of device-passes was significantly lower for the SAVE group in the carotid T (median 2, IQR 1–3 vs median 3, IQR 2–5; *p* < 0.001) but not in the M1 population (median 1, IQR 1–2 vs median 2, IQR 1–3; *p* = 0.133). In the M2 population no difference was observed. We observed no significant difference in the rate of ENT (3.0% vs 12.%, *p* = 0.57) after correction for multiple testing.

**Table 4:** Secondary outcomes

Endpoint	Overall	ADAPT	SAVE	*p* Value
First-pass mTICI ≥2b	48.5%	33.3%	59.6%	**0.019**
Carotid T	27.3%	11.5%	41.4%	0.323
M1 segment	57.4%	41.7%	67.2%	0.361
M2 segment	63.6%	60.0%	66.7%	1
First-pass mTICI ≥2c	27.5%	16.7%	35.4%	0.171
Carotid T	16.4%	0.0%	31.0%	**0.038**
M1 segment	36.2%	30.6%	39.7%	1
M2 segment	18.2%	10.0%	25.0%	1
Device-passes (Median, IQR)	–	2, 2–3	1, 1–2	**< 0.001**
Carotid T	–	3, 2–5	2, 1–3	**< 0.001**
M1 segment	–	2, 1–3	1, 1–2	0.133
M2 segment	–	1, 1–2	1, 1–2	1
ENT	12 (7)	9 (12.5)	3 (3)	0.570
Carotid T	6 (10.9)	6 (23.1)	0 (0)	0.152
M1 segment	5 (5.3)	3 (8.3)	2 (3.4)	1
M2 segment	1 (4.5)	0 (0)	1 (8.3)	1
mRS on 90 d (Median, IQR)		3, 2–5	3, 1–6	1
Post interventional SAB	27 (15.8)	15 (20.8)	12 (12,1)	1
sICH	8 (4.8)	3 (4.2)	5 (5.2)	1

*ENT* embolies to new territory, *IQR* interquartile Range, *mRS* modified Rankin Scale, *sICH* symptomatic intracranial hemorrhage

## Discussion

In this study, we report the angiographic results and time-metrics from our center in the timeperiod from January 2014 to September 2017. Our data supports the notion that a combined approach such as SAVE is more efficient in the treatment of LVO in the anterior circulation than ADAPT: The rates of combined mTICI 2b, 2c and 3 (93,5% vs 75.0%), combined mTICI 2c and 3 (54.5% vs. 33.3%) were all higher for the SAVE group. Simultaneously, the SAVE approach did not result in longer groin to reperfusion times.

For SAVE the rate of successful reperfusion (mTICI 2b or better) parallels other publications regarding combined approaches such as CAPTIVE, PROTECT and ARTS, who report rates of mTICI 2b or better of 100 and 98% respectively [8, 9, 12]. However the frequency of mTICI 2c or better (54.5%) for SAVE was lower than in the CAPTIVE trial (79.5%) and in unpublished work from our group. This discrepancy can be attributed to the real-world design of this study. Contrary to the ASTER, CAPTIVE and ARTS trial [7–9] we did not exclude tandem occlusions (19.9% of the cases) from our analysis. Also five of eight interventionalists did perform only 6 procedures per technique or less. It can be assumed, that the angiographic results will improve with more training. Furthermore the frequency of carotid T occlusions was higher (32.2%) compared to the ASTER Trial (~ 10%) and ~ 23% in the ADAPT FAST study [21]. These deviations might also explain the lower frequency of successful reperfusion (defined as mTICI≥2b) in our study for ADAPT (75.0%) compared to the ASTER Trial (85,4%) [7] and self-reported single-center trials (90–95%) [18, 21].

This interpretation is supported by our data, since after stratification by the occluded vessel it becomes evident, that in case of carotid T occlusion SAVE is superior to ADAPT in terms of archiving successful reperfusion (mTICI≥2b in 93.1% vs 65.4%; *p* = 0.025) and near complete reperfusion (mTICI≥2c in 55.2% vs 15.4%; *p* = 0.010). In the M1 Segment our reperfusion success with ADAPT (83.3%) is comparable to the ASTER Trial [7]. This is in-line with previous reported data, that an occlusion of the carotid T is an independent predictor for worse angiographic outcomes in ADAPT [22]. Possible explanations for this observation are that carotid T occlusions have higher thrombus burden and not optimal ratios of vessel size to aspiration-catheter diameter [22]. This is in line with the notion, that the SAVE technique might be superior in case of a larger thrombus, since it can be squeezed better between the stent retriever and the distal aspiration catheter. For the M1 segment no significant effect was observed in the data, however the high OR for archiving mTICI≥2b (8.325) indicate that this was most likely due to the small sample size. In case of the M2 segment the effect size was very small, although it has to be noted, that the small sample size (*n* = 22; 10 ADAPT, 12 SAVE) severely reduces our ability to draw a conclusion. However the anatomy might not be optimal for the placement of the stent-retriever, since the diameter of the vessel is very narrow. Work from our group showed a correlation between vessel diameter and reperfusion results, especially for SAVE [23]. Furthermore it cannot be placed distal enough to ensure optimal clot control. Also the push maneuver is not so efficient, if the clot is more distal. In addition a smaller thrombus might fit easier in the aspiration catheter.

Regarding the utilization of an additional BGC as in PROTECT [12], we opted against this approach, as in our mind proximal aspiration through the guide-catheter and distal aspiration through the aspiration-catheter allows sufficient control of the thrombus, especially in combination with the very distal placement of the stent-retriever, which is reflected in the very low number of ENT (3.0%). However it has to be noted, that the PROTECT plus study with very high rates of first-pass mTICI3 (59.4%) [13] raised the question again, if this notion is correct or if a BGC can indeed improve thrombus control even in combined approaches.

From an economic standpoint combined approaches such as SAVE result in increased costs due to the simultaneous use of multiple devices [24, 25]. However, this argument is weakened by the fact, that in 30% of the ADAPT cases a rescue maneuver with a stent-retriever was necessary. This is in-line with the ASTER Trial, in which 32,8% of the first-line ADAPT cases needed a rescue maneuver with a stent retriever. Other studies reporter frequencies of 22% [21] to 34% [6]. Furthermore, if a rescue maneuver was necessary the reperfusion times were prolonged significantly [21].

Although SAVE resulted in significant better reperfusion scores and significant less device- passes, we were not able to find an effect on clinical outcome data such as 90 days mRS. This was not expected, since multiple other studies showed a tight correlation between angiographic success defined as mTICI 2c or better and clinical outcome [26–28]. Furthermore in-vitro work by Chueh et al. demonstrated a correlation between clot fragmentation leading to downstream embolic events and the number of attempts [29]. Moreover a recent network meta-analysis of randomized trials revealed a potential benefit of first-line stent-retriever over aspiration [30]. Also in the ASTER Trial a slight trend for a higher frequency of functional independence (mRS ≤ 2 at 90 days) was observable with rates of 50% in the stent-retriever group and 45% in the aspiration group [7]. Possible explanation for our failure to detect an effect on clinical outcome is the heterogeneity of our treatment group. Most ADAPT cases were in the time from 2014 to 2016. Since then the selection criteria for EVT were widened in accordance to new guidelines. Secondly in the SAVE group more patients were not directly transferred to our hospital resulting in longer symptom to admission times. Furthermore the frequency of carotid T occlusions was very high (~ 30%), especially in transferred patients, which is a predictor of worse outcome by itself as it was shown recently in the THRACE trial [31].

One major limitation of this study is the retrospective, observational design which is prone to selection biases. In addition to the selection bias it has to be noted, that the technique was chosen by the interventionalist, which might has introduced bias to this work, since their experience with the different techniques was not equal. Secondary analysis which is not included in this paper showed however, that the results were not affected by excluding the inexperienced interventionalist (less than 10 cases). Also workflow changes might distort the picture since a new triage paradigm was introduced in the observed period. Furthermore the number of transferred patients differed between both groups as mentioned above.

## Conclusion

Embolectomy using SAVE has the potential to improve angiographic outcomes compared to ADAPT, especially in the carotid T. However, randomized control studies have to be conducted to elucidate the effect of a combined approach on outcome.

## Abbreviations

ADAPT: A Direct Aspiration First Pass Technique
AIS: Acute ischemic stroke
ARTS: Aspiration-Retriever Technique for Stroke
BGC: Balloon-Guiding Catheter
CAPTIVE: Continuous Aspiration Prior To Intracranial Vascular Embolectomy
CI: Confidence intervals
ENT: Emboli to new territory
EVT: Endovascular treatment
IQR: Interquartile range
ITT: Intention to treat
LVO: Large vessel occlusion
mRS: Modified Rankin Scale
mTICI: Modified treatment in cerebral ischaemia
PROTECT: PRoximal balloon Occlusion TogEther with direCt Thrombus aspiration during stent retriever thrombectomy
SAVE: Stent-retriever Assisted Vacuum-locked Extraction
sICH: Symptomatic intracranial Hemorrhage
